# Precut disc of lyophilized amniotic membrane for recurrent full thickness macular hole management

**DOI:** 10.1007/s00417-025-06955-x

**Published:** 2025-09-11

**Authors:** Rita Serra, Thibaud Mathis, Yasmine Serrar, Lucas Sejournet, Giacomo Boscia, Francesco Boscia, Antonio Pinna, Laurent Kodjikian

**Affiliations:** 1 Service d’Ophtalmologie, Hôpital de la Croix-Rousse, Hospices Civils de Lyon, Lyon, 69004 France; 2 Department of Medicine, Surgery and Pharmacy, Ophthalmology Unit, University of Sassari, Sassari, Italy; 3 Istituto di Ricerca Genetica e Biomedica (IRGB), CNR, Cittadella Universitaria di Cagliari, Monserrato, 09042 CA Italy; 4 Laboratoire MATEIS, UMR-CNRS 5510, INSA, Université Lyon 1, Villeurbanne, 69100 France; 5 Department of Translational Biomedicine Neuroscience, University of Bari “Aldo Moro”, Piazza Giulio Cesare, 11, Bari, Italy

**Keywords:** Full thickness macular hole, Lyophilized amniotic membrane transplantation, Pars plana vitrectomy

## Abstract

**Purpose:**

To assess the efficacy of precut disc of lyophilized amniotic membrane (LAM) in the management of recurrent full thickness macular hole (FTMH), already treated with pars plana vitrectomy (PPV), internal limiting membrane peeling, and gas tamponade.

**Methods:**

Twenty-seven eyes of 27 patients with recurrent FTMH were included in this retrospective study and treated with 25G PPV, LAM graft (Visio Amtrix, TBF, Mions, France) and 20% SF6 gas tamponade. Spectral domain optical coherence tomography (SD-OCT) was used to assess anatomic FTMH closure 1 month postoperatively. Best corrected visual acuity (BCVA) was measured preoperatively and 1, 3, 6 and 12 months after surgery.

**Results:**

Twelve eyes presented large idiopathic FTMHs, 12 showed large FTMH associated with high myopia and the remaining 3 had large FTMH and retinal detachment in highly myopic eyes. Mean minimum FTMH diameter at baseline was 782±341.17 μm and mean BCVA was 1.02±0.63 LogMAR. Postoperatively, anatomical FTMH closure was achieved in 23/27 (85.2%) eyes one month after the surgery. Mean BCVA progressively improved during the follow-up period, reaching 0.55±0.42 LogMAR (*p* = 0.04) at 12-months follow-up. No adverse events were registered during follow-up.

**Conclusions:**

Results suggest that epiretinal graft of LAM precut discs is a safe and effective surgical procedure for large and recurrent FTMH treatment, leading to significant BCVA improvement.

**Supplementary Information:**

The online version contains supplementary material available at 10.1007/s00417-025-06955-x.

## Key messages

**What is known:**

- Encouraging anatomical results have been reported by using human amniotic membrane (hAM) graft for treatment of recurrent idiopathic or complex full thickness macular hole (FTMH) in eyes with co-morbidities such as high myopia with or without retinal detachment.
- Recently, liophylized hAM (LAM) patches, showing physical, biological and structural properties equivalent to classical hAM, have become available for clinical use. LAM may be stored at room temperature for long periods without deterioration, and its transportation is easy and inexpensive contrary to hAM, stored by cryopreservation, a deep freezing facility, expensive and not always available.

**What is new:**

- This study revealed that epiretinal graft of LAM precut discs resulted in an 85.2% rate of anatomical FTMH closure one month after the surgery and was associated with a significant mean best corrected visual acuity improvement at 12-months follow-up in large and recurrent cases.
- Our results disclosed that epiretinal graft of LAM precut discs is a safe and efficace surgical procedure for management of recurrent large FTMH in the idiopathic and complex cases.

**Supplementary Information:**

The online version contains supplementary material available at 10.1007/s00417-025-06955-x.

## Introduction

Human amniotic membrane (hAM) is the innermost layer of the placenta, directly in contact with the amniotic liquid [1]. It appears as a semi-transparent tissue with a thickening ranging from 0.02 to 0.05 mm. hAM consists of three different layers, including the epithelium, basement membrane, and avascular stroma. It is similar to the conjunctiva and cornea in terms of collagen structure and the production of several biological factors with anti-inflammatory, anti-angiogenetic and anti-fibrotic action [1, 2].

Originally used for skin transplantation [3], hAM use has become a common procedure in ophthalmology, including conjunctival reconstruction, scleral perforation management, and glaucoma surgery [4, 5]. Recently, encouraging results have been reported for a variety of retinal diseases, such as age-related macular degeneration, retinal breaks, optic pit maculopathy, and recurrent full thickness macular hole (FTMH) [6, 7].

However, depending on the Center, hAM is not always easily available and must be scheduled in advance. Furthermore, the current surgical techniques used for FTMH management present some limitations, mainly due to the difficulties in hAM patch handling in the vitreous cavity, as well as its positioning on the FTMH, with the potential risk of injury to foveal retinal pigment epithelium (RPE). FTMH surgery itself may be complicated by retinal tears, retinal detachments, phototoxicity as well as peripheral field defects and side-effects secondary to post-operative face-down positioning (i.e., skin breakdown, pulmonary embolism, ulnar nerve neuropathy with ulnar paresthesia and muscle atrophy) [8, 9].

Recently, precut liophylized hAM (LAM) patches, showing physical, biological and structural properties equivalent to classical hAM, have become available for clinical use. LAM may be stored at room temperature for long periods without deterioration, and its transportation is easy and inexpensive [10, 11].

The use of LAM in ocular surface surgery has already been described, but very few data are available for recurrent FTMH surgery [12]. Moreover, recent advances in LAM design, with precut patches of specific diameters, have improved surgery with easier membrane handling.

Therefore, the purpose of this case series was to assess the efficacy of precut LAM discs (Visio Amtrix, TBF, Mions, France) in the management of recurrent FTMH, already treated with pars plana vitrectomy (PPV), internal limiting membrane (ILM) peeling, and gas tamponade.

## METHODS

The present study was conducted in accordance with the code of ethics of the World Medical Association (Declaration of Helsinki) for research involving human subjects. All participants gave their informed consent to study protocols, which were approved by Ethics Committee of the French Society of Ophthalmology, (IRB 00008855 Société Française d’Ophtalmologie IRB#1).

### Patients’ selection

All patients with recurrent FTMH treated surgically with the use of LAM were included in this retrospective study, conducted at the Ophthalmology Department of the Croix-Rousse University Hospital, Lyon, France, between January 2019 and September 2022.

Inclusion criterion was the presence of recurrent FTMH, associated or not to retinal detachment, already treated with at least one prior surgery with PPV, ILM peeling, and gas tamponade.

Exclusion criteria were the presence of any history of diabetic retinopathy, uveitis, retinal vascular disease, or ocular trauma.

All patients enrolled underwent a complete ophthalmological examination, including best corrected visual acuity (BCVA) measured on Snellen chart and converted in LogMAR for analyses, Goldmann applanation tonometry, indirect fundus ophthalmoscopy, and spectral domain optical coherence tomography (SD-OCT Spectralis; Heidelberg Engineering, Germany) for FTMH analysis, both pre- and post-operatively (at 1, 3, 6, and 12 months of follow-up). A previously validated tool (Heidelberg Eye Explorer software; Heyex, Version 1.7.0.0; Heidelberg Engineering) was used to measure FTMH diameter.

### Surgical technique

All the surgical procedures were performed by the two senior vitreoretinal surgeons.

Standard three-port 25-gauge (G) transconjunctival sutureless PPV was performed using Constellation vitrectomy machine (Alcon, Fort Worth, TX, USA), under local anesthesia (peribulbar block).

Triamcinolone acetonide was injected into the vitreous cavity to verify and complete vitreous removal if necessary. Thereafter, vital dye (ILM-Blue, DORC International, Zuidland, Netherlands) was sprayed onto the posterior retina to stain ILM residuals, which could be removed, if necessary, by intraocular forceps. In case of recurrent FTMH associated with retinal detachment, perfluorocarbon liquid was used to help drainage of subretinal fluid *ab interno*. Moreover, peripheral retinal tears were treated with cryopexy.

Then fluid-air exchange was performed, leaving a thin fluid layer on the FTMH to help LAM graft positioning. One LAM patch of 3 to 5 mm of diameter, depending on FTMH size, was flipped and rolled inside the intraocular forceps and thus inserted through the temporal valved trocar into the vitreous cavity to be gently placed in the epiretinal space at level of the FTMH.

A drop of a viscoelastic substance (Viscoat, Alcon) was placed over the LAM graft to limit the risk of dislocation. Fluid-air exchange was carefully completed using a silicone tip backflush cannula to ensure LAM patch adhesion to the underlying inner retina.

Air-gas exchange using 20% sulfur hexafluoride (SF6) was performed as endotamponade and injected into the vitreous cavity. Patients were instructed to maintain face-down positioning for up to 3 days after surgery.

### Statistical analysis

The results of descriptive analysis are reported as numbers and percentages for categorical variables and as means ± standard deviation (SD) for quantitative variables. After testing the data distribution for normality, Mann-Whitney test was used, as appropriate. The statistical significance level was set at *p* < 0.05. The study data were analyzed using the Statistical Package for Social Sciences version 20.0 for Mac (IBM, Chicago, IL).

## RESULTS

Twenty-seven eyes of 27 patients (11 men, 16 women; mean age 62.16±12.16 years) with recurrent FTMH previously treated with PPV and ILM peeling were included.

Twelve (44.4%) FTMHs were idiopathic, twelve (44.4%) were associated with high myopia and the remaining three (11.2%) were associated with both high myopia and retinal detachment.

Overall, the mean minimum FTMH diameter at baseline was 782±341.17 μm (range 475–1495 μm). Specifically, this was 779.88±322.02 μm in idiopathic FTMH eyes, 784.40±372.09 μm in the highly myopic FTMH group and 807.33±232.42 μm in the eyes with high myopia and retinal detachment. All baseline characteristics are summarized in Table 1.

**Table 1:** Patient characteristics at baseline

	Idiopathic FTMH group *N* = 12	FTMH in high myopic group *N* = 12	High Myopia FTMH with RD group *N* = 3
Gender, n (%) - Male - Female Mean age, years + SD Mean axial length + SD (mm) Mean FTMH size, µm (SD) Mean BCVA, LogMAR (SD)	5 (41.7%) 7 (58.3%) 69.8 + 13.18 23.11 + 0,98 779.88 + 322.02 1.04 + 0.66	5 (41.7%) 7 (58.3%) 55.75 + 7.88 30.88 + 3.02 784.40 + 372.09 1.00 + 0.64	1 (25%) 2 (75%) 62.33 + 10.69 29.30 + 1.76 807.33 + 232.42 1.60 + 0.69

BCVA: Best-corrected visual acuity; FTMH: full thickness macular hole; SD: standard deviation

One month after the surgery, anatomical FTMH closure was achieved in 23/27 (85.18%) eyes; specifically, in 10/12 (83.3%) eyes with the idiopathic form, in 10/12 (83.3%) with high myopia, and in 3/3 (100%) highly myopic eyes complicated by retinal detachment.

**Fig. 1:**
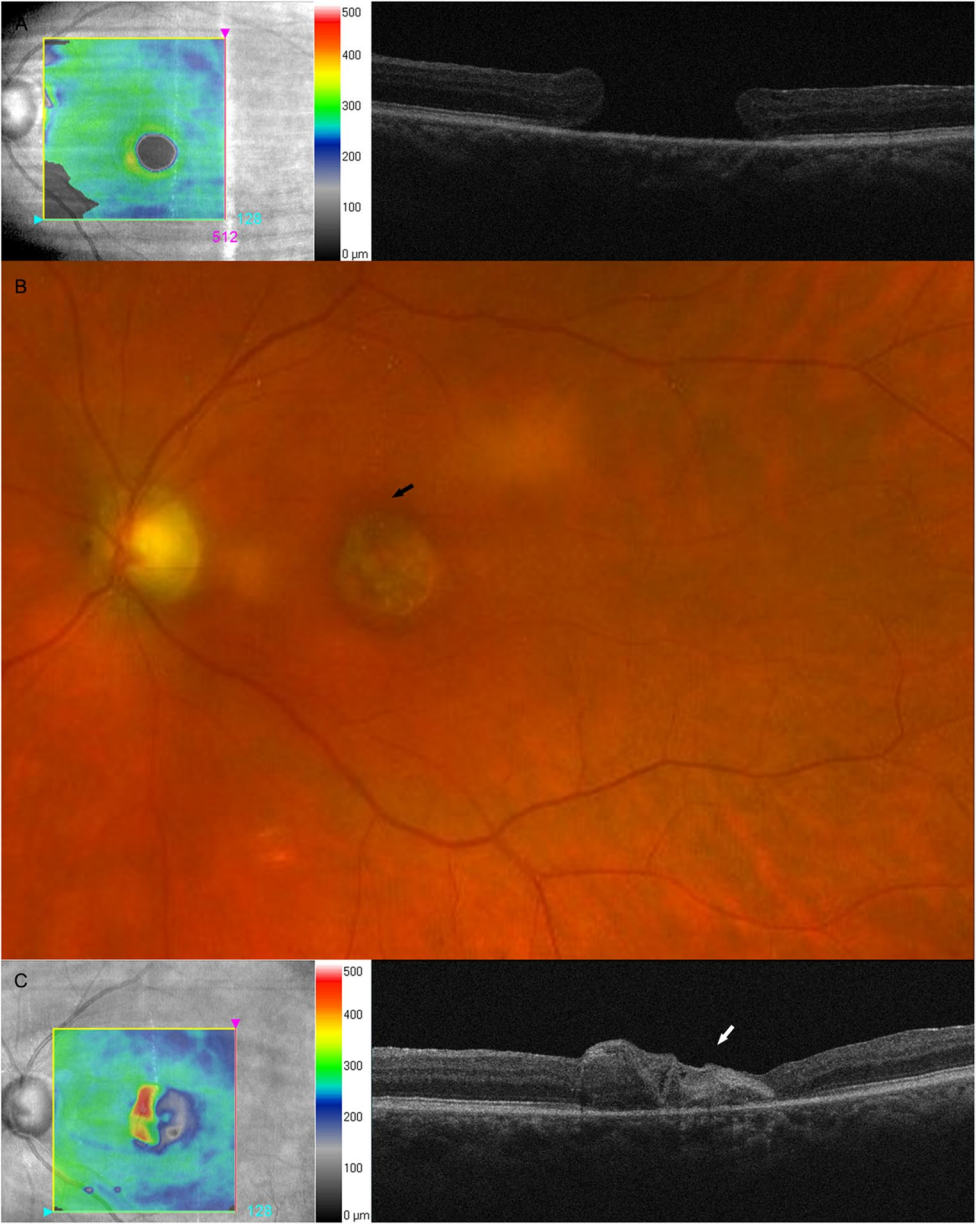
Multimodal imaging of recurrent idiopathic full thickness macular hole (FTMH) patient successfully treated by graft of precut disc of lyophilized amniotic membrane (LAM)

Figure 1 shows a case of recurrent FTMH closed by LAM graft.

**(A)** Preoperatively, simultaneous infrared and optical coherence tomography (OCT) scan showed recurrent full thickness macular hole (FTMH). **(B)** Twelve months after surgery, fundus photograph showed the epiretinal graft of precut LAM (black arrow). (**C**) simultaneous infrared and OCT scan revealed recurrent FTMH closed by hyper-reflective tissue corresponding to LAM graft (white arrow).

**Fig. 2:**
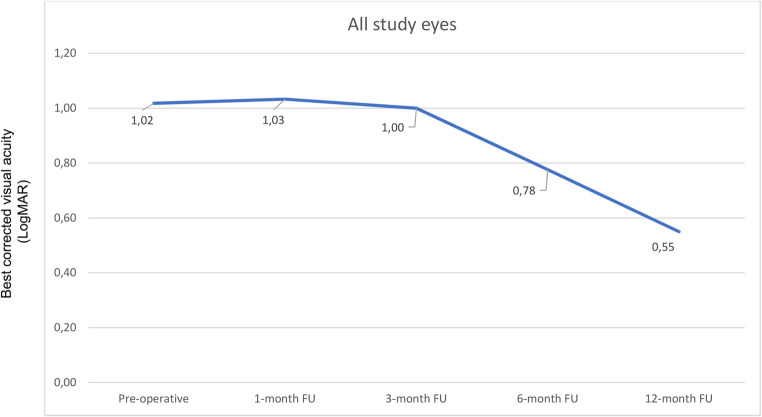
Graph showing functional outcome during one year of follow-up (FU)

FTMH closure was not achieved in only 4 out of 27 eyes; therefore it was not possible to establish a correlation to the location of LAM over FTMH. LAM graft dislocation was observed in all four cases later than 3 days postoperatively, when face-down position was finished. Overall, one year after surgery mean BCVA was significantly higher than preoperative BCVA (1.02±0.63 LogMAR vs. 0.55±0.42 LogMAR; *p* = 0.04). Similarly, mean BCVA progressively improved after surgery during the entire follow-up period (Fig. 2), and it was found to be higher in all study groups (Table 2). No ocular adverse events such as retinal tears, retinal detachment and RPE damage were noted during follow-up.

**Table 2:** Best corrected visual acuity, measured in logmar, at baseline, 1,3 6 and 12 months of follow-up

	BCVA baseline	BCVA 1 month FU	BCVA 3 months FU	BCVA 6 months FU	BCVA 12 months FU
Idiopathic FTMH eyes	1.04 ± 0.42	0.98 ± 0.62	0.99 ± 0.53	0.64 ± 0.39	0.50 ± 0.45
High myopia FTMH eyes	1.00 ± 0.64	1.1 ± 0.47	1.01 ± 0.58	0.86 ± 0.63	0.60 ± 0.42
High myopia FTMH eyes with retinal detachment	1.60 ± 0.69	1.30 ± 0.7	1.33 ± 0.65	1.37 ± 0.60	1.00 ± 0.50

BCVA: Best-corrected visual acuity; FTMH: Full thickness macular hole; FU: Follow-up

## DISCUSSION

In this case series, LAM graft was performed to treat recurrent FTMH in eyes with the idiopathic form, high myopia or retinal detachment associated with high myopia.

In the last years, there has been an important improvement in the surgical techniques for FTMH management, leading to its anatomical closure rate over 90% of idiopathic cases [13–15].

However, the presence of co-morbidities (e.g., high myopia, sometimes complicated by retinal detachment), makes the surgical success more difficult to achieve. Indeed, in such cases, the anatomical closure rate of FTMH has been reported to reach 50% [16, 17].

Recently, surgical techniques for FTMH management have focused upon the search for a substrate able to seal the hole and promote retinal proliferation and restoration.

Specifically, Lai et al. [18] have reported a closure rate of 96% by using autologous blood in a pool of 27 FTMH eyes with high myopia.

In another investigation, Morizane et al. [19] showed that autologous ILM flap transplantation was a safe and effective technique in 9 out of 10 eyes, with a visual gain in 8 of them. By using this method, Chen et al. [20] described a 100% closure rate in a cohort of 13 FTMH eyes with retinal detachment. However, it is important to emphasize that it is difficult to collect an ILM flap with a size appropriate for large FTMH in highly myopic eyes, which had already undergone ILM peeling. Furthermore, the ILM flap thickness makes the surgical maneuver to place the flap in the hole difficult and time consuming.

In another case series of 6 FTMHs with retinal detachment, Wu et al. [21] obtained an anatomical closure rate of 66.7% by using a retinal autograft, a technique characterized by difficult surgical steps and high risk of complications (i.e., retinal detachment, vitreous hemorrhage).

Overall, although the results of the above-mentioned studies seem to be encouraging [19–21], the surgical techniques used are difficult. Moreover, the samples evaluated are too small to draw any robust conclusion on this topic.

Recently, Rizzo et al. [7] have shown excellent anatomical results using hAM graft for FTMH in highly myopic eyes with or without retinal detachment. However, the hAM patch needs to be pre-cut and adapted to the FTMH size to allow its insertion into the hole in the sub-retinal space. Interestingly, by using this technique, Caporossi et al. [22] have reported a 100% closure rate in 10 FTMH eyes with high myopia and retinal detachment. However, patch handling and positioning into the hole may cause injury to the foveal RPE, thereby negatively affecting visual recovery. To this regard, Tsai et al. [23] have reported the occurrence of parafoveal atrophy in 4 out of 10 FTMH eyes with high myopia which had undergone hAM graft into the sub-retinal space.

Moreover, hAM used in the above-mentioned published studies were stored by cryopreservation, currently considered the ideal storage method to guarantee the biological hAM properties. However, this method requires a deep-freezing facility (up to −80 °C), which is expensive and not always available, especially in underdeveloped countries. Recently, the lyophilization process has been proposed as an alternative method for biological tissue storage. By this technique, water is removed by sublimation, thus preventing biochemical reactions that can cause its deterioration.

As far as we known, Garcin et al. [10] were the first to describe LAM graft for recurrent FTMH management. Specifically, they used LAM patches that needed to be punched as 3- or 4-mm diameter discs, depending on the FTMH size, before surgery. Unlike the technique by Rizzo et al. [7], Garcin et al. [12] proposed to place the LAM graft on the fovea, rather than in the hole at the level of the sub-retinal space. This approach seems to be easier and with a lower risk of damaging the LAM patch during manipulation or causing retinal injury during the maneuvers necessary to its placement into the sub-retinal space, as in the method described by Rizzo et al. [7].

In the study by Garcin et al. [12] FTMH closure was achieved in 8 out of 10 eyes, five of which had a regmatogenous retinal detachment.

Unlike Garcin et al. [12], in our case series, we used pre-cut discs of LAM, rather than LAM patches trephined during surgery. Specifically, we used pre-cut LAM discs with a 3- or 5-mm diameter, depending on the FTMH size, placed over the fovea to treat 27 eyes with recurrent FTMH eyes. No adverse events were recorded and anatomical FTMH closure was achieved in 10/12 eyes with the idiopathic form, in 10/12 eyes with high myopia and in all the three highly myopic eyes with retinal detachment.

One month postoperatively, SD-OCT analysis revealed that in twenty-three (85.18%) eyes the LAM patch was centered on the fovea and FTMH resulted to be closed. There was progressive improvement of mean BCVA, which reached 0.55±0.42 LogMAR (*p* = 0.04) at 12-months follow-up.

Histological hAM analysis has revealed that its extracellular matrix is reach of hyaluronic acid, collagen, laminin, fibronectin, and proteoglycans. hAM making is characterized by good cell adhesion and peculiar mechanical properties (permeability, stability, elasticity, flexibility, resorbability) [24–26].

LAM, obtained by sublimation technique, has the same biological properties as hAM and may be easily stored at room temperature for long periods with no deterioration. It is a “ready to use” product, rich of growth factors, providing a natural scaffold on which epithelial cells may migrate, grow, and then promote FTMH closure.

Specifically, it is likely that LAM disc may rappresent a scaffold inducing the formation of a glial bridge of microfibrils between FTMH edge contributing its anatomical closure. Consequently, the proliferation process may fill in the cystoid spaces, thus promoting restoration of the external retinal layers, explaining a functional improvement secondary to anatomical FTMH closure. This theory is supported by transmission electron microscopy analysis of cultured human RPE (hRPE) cells on hAM, as reported by Capeans et al. [27] These authors observed that hRPE cells seeded onto hAM exhibited elongation of their basal membrane, which became embedded in the hAM, with subsequent cell adhesion and growth on epithelium-free hAM. Thereafter, hRPE cells were able to proliferate while maintaining their epithelial phenotype, with typical morphological and pigment characteristics. These cells formed tight colonies that developed into a monolayer with cuboidal to spheroidal morphology. This histological phenomenon is thought to be promoted by amniotic stroma, which secretes growth factors and express adhesion molecules facilitating cell proliferation.

This case series has some limitations, including the small sample size and its retrospective nature. The most important limitation is that this was not a randomized prospective investigation in which the patients were randomly distributed between a study group and a control group. Nevertheless, we are unaware of any former study using precut discs of LAM for the management of recurrent, large idiopathic or complex FTMH.

In conclusion, results suggest that epiretinal graft of LAM precut discs is a safe and effective surgical procedure for large and recurrent FTMH treatment, leading to significant BCVA improvement. Future large case-control studies are necessary to confirm our findings.

## Supplementary Information

Below is the link to the electronic supplementary material.

Supplementary Material 1 (DOCX. 3.78 MB)
