# Supplementary material for: Precut disc of lyophilized amniotic membrane for recurrent full thickness macular hole management
Source: Graefes Arch Clin Exp Ophthalmol. 2025 Sep 11;263(12):3365–72. doi: 10.1007/s00417-025-06955-x (PMC12886322; doi:10.1007/s00417-025-06955-x)
Supplement: Supplementary file 1 — Supplementary Material 1 (DOCX. 3.78 MB) [file 417_2025_6955_MOESM1_ESM.pdf]

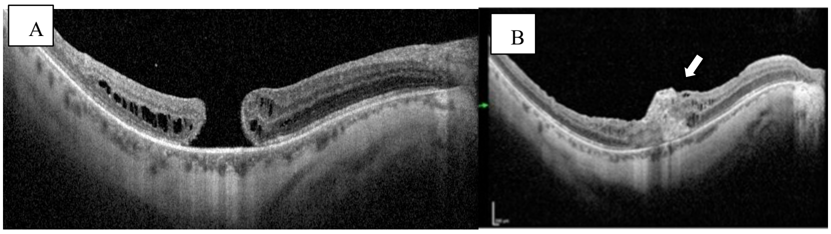


Case 1. (A) Full thickness macular hole in high myopic eye successfully treated with LAM graft (B). Note the LAM in B (white arrow in B).

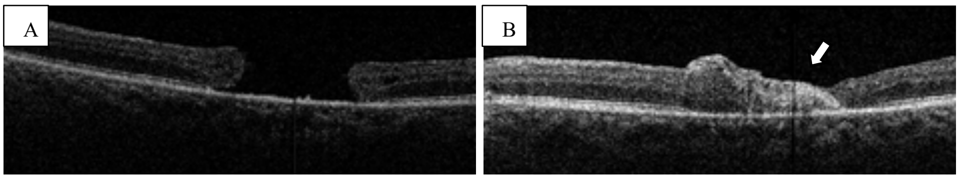


Case 2. (A) Full thickness macular hole successfully treated with LAM graft (B). Note the LAM in B (white arrow in B).

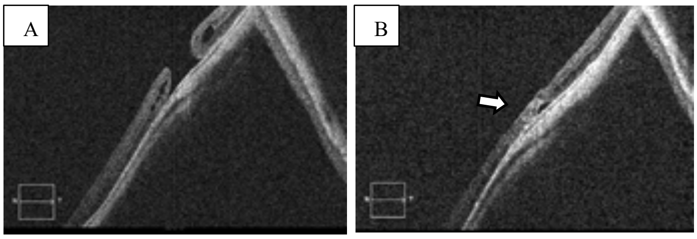


Case 3. (A) Full thickness macular hole in high myopic eye successfully treated with LAM graft (B). Note the LAM in B (white arrow in B).

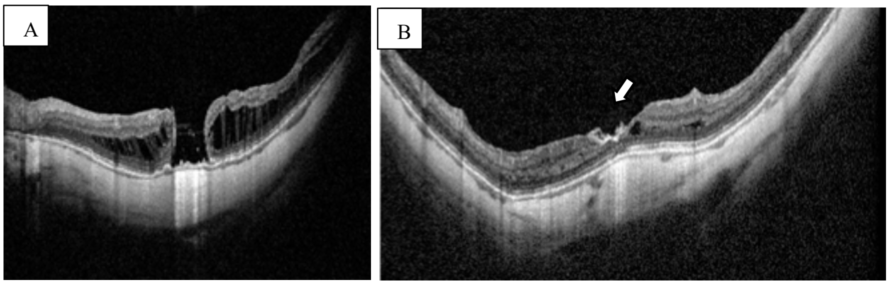


Case 4. (A) Full thickness macular hole in high myopic eye successfully treated with LAM graft (B). Note the LAM in B (white arrow in B).

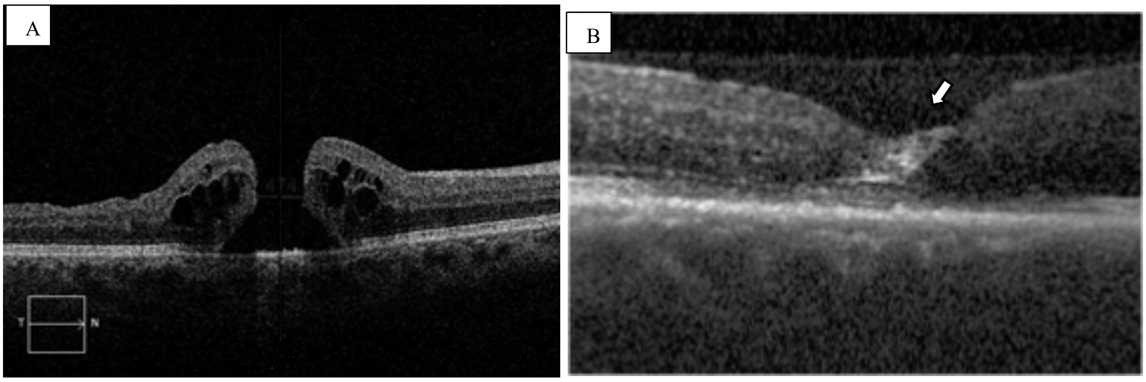


Case 5. (A) Full thickness macular hole successfully treated with LAM graft (B). Note the LAM in B (white arrow in B).

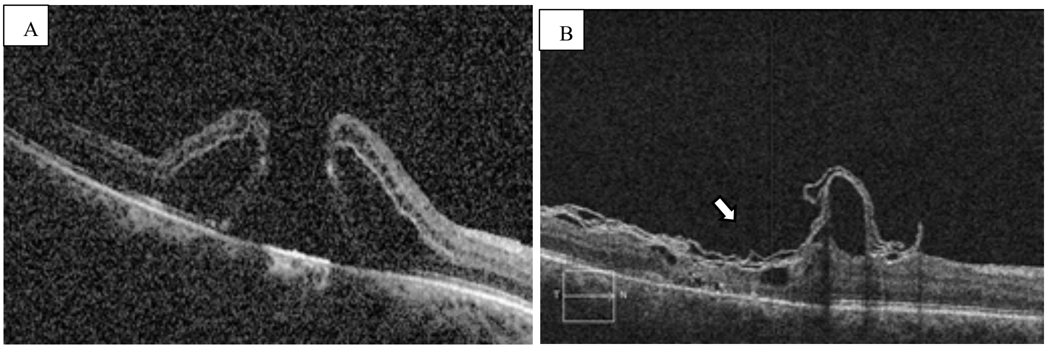


Case 6. (A) Full thickness macular hole successfully treated with LAM graft (B). Note the LAM in B (white arrow in B)

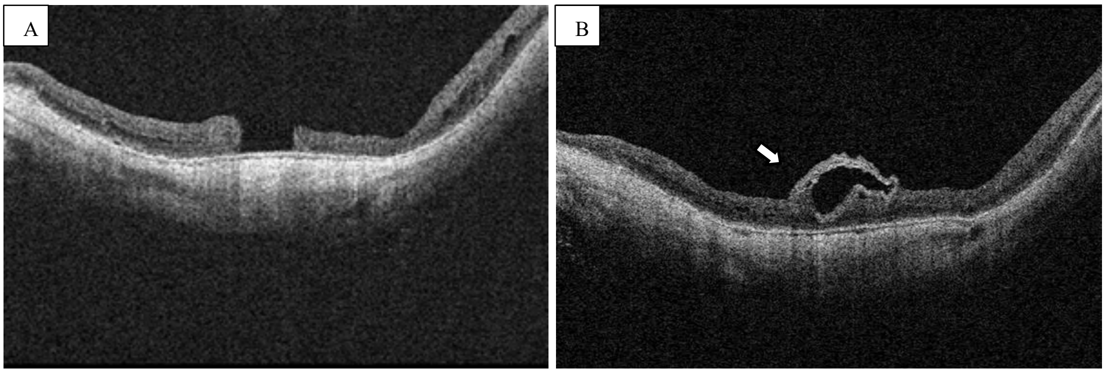


Case 7. (A) Full thickness macular hole in high myopic eye successfully treated with LAM graft (B). Note the LAM in B (white arrow in B).

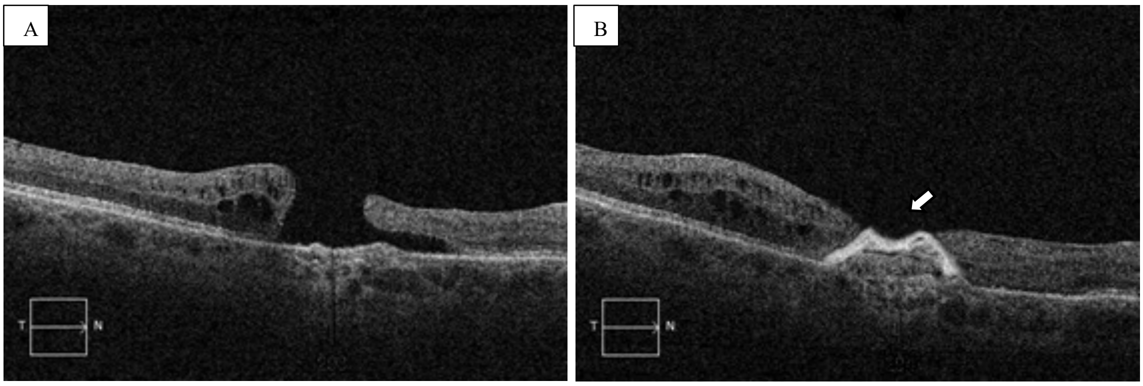

Case 8. Full thickness macular hole successfully treated with LAM graft (B).  Note the LAM in B (white arrow in B).

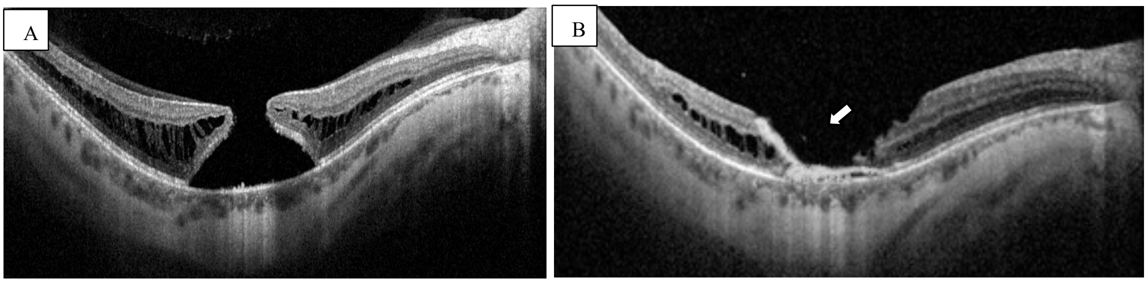


Case 9. Full thickness macular hole successfully treated with LAM graft (B). Note the LAM in B (white arrow in B).

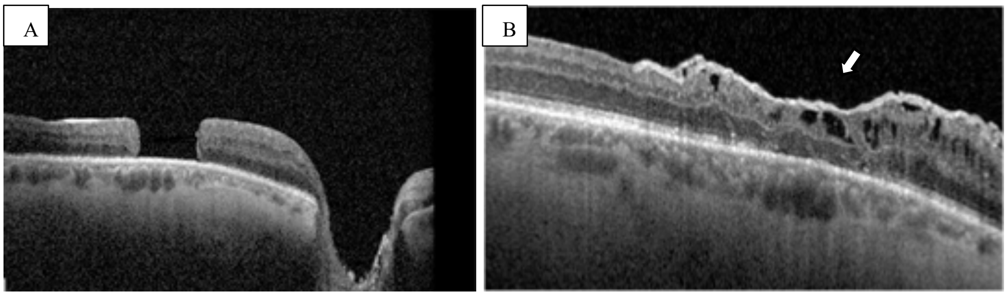


Case 10. (A) Full thickness macular hole successfully treated with LAM graft (B). Note the LAM in B (white arrow in B)

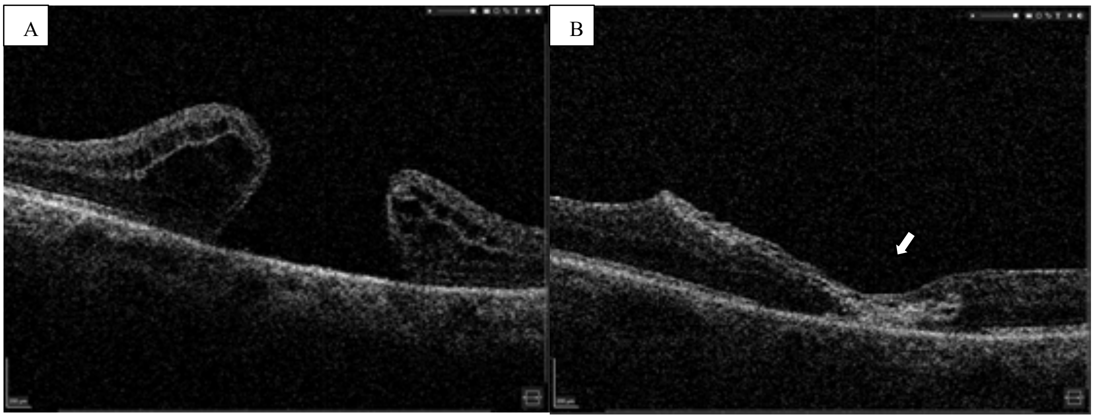


Case 11. (A) Full thickness macular hole successfully treated with LAM graft (B). Note the LAM in B (white arrow in B)

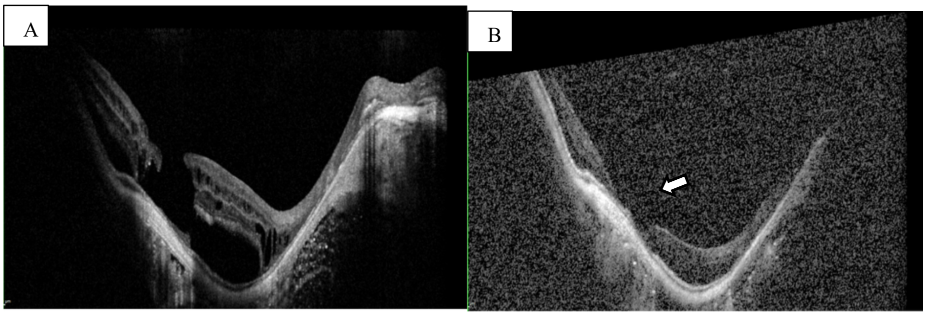

Case 12.-Full thickness macular hole with high myopia and retinal detachment successfully treated with LAM graft (B). Note the LAM in B (white arrow in B)

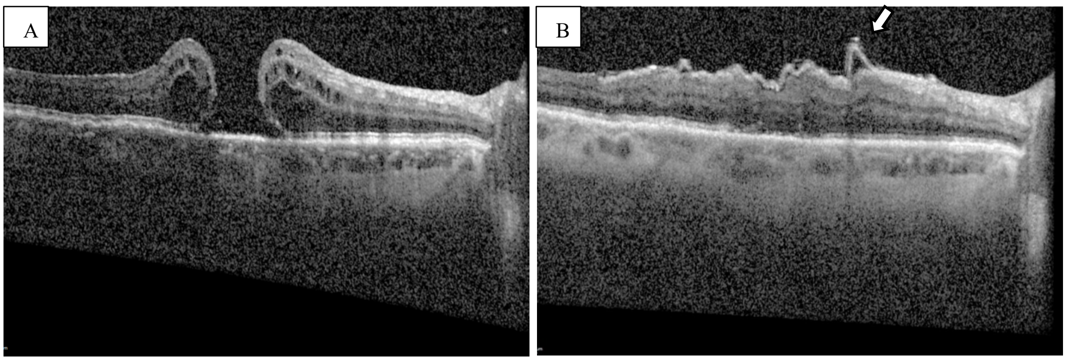


Case 13. (A) Full thickness macular hole successfully treated with LAM graft (B). Note the LAM in B (white arrow in B).

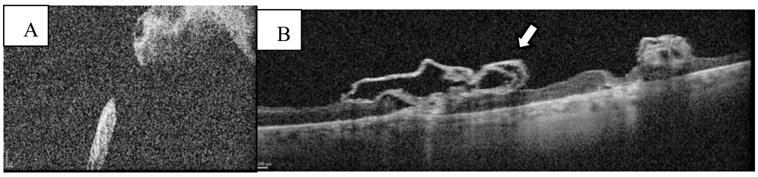


Case 14. (A) Full thickness macular hole with high myopia and retinal detachment, successfully treated with LAM graft (B). Note the LAM in B (white arrow in B).

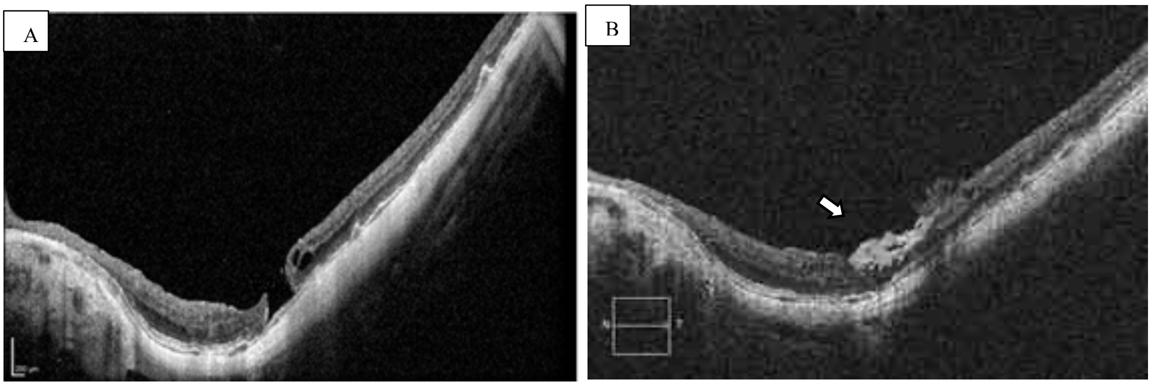


Case 15. (A) Full thickness macular hole in high myopic eye successfully treated with LAM graft (B). Note the LAM in B (white arrow in B).

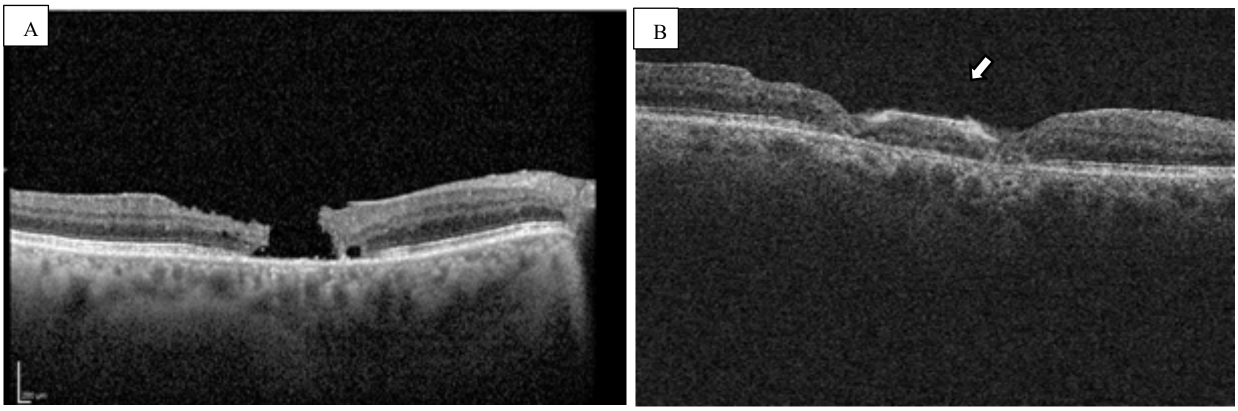


Case 16. Full thickness macular hole successfully treated with LAM graft (B). Note the LAM in B (white arrow in B).

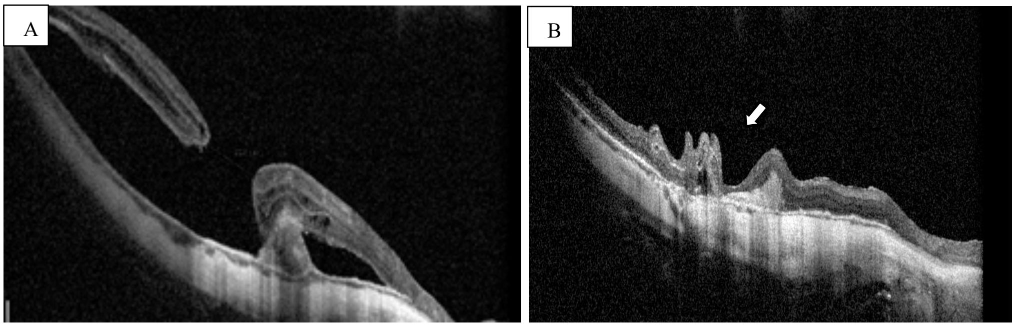


Case 17. (A) Full thickness macular hole with high myopia and retinal detachment successfully treated with LAM graft (B). Note the LAM in B (white arrow in B).

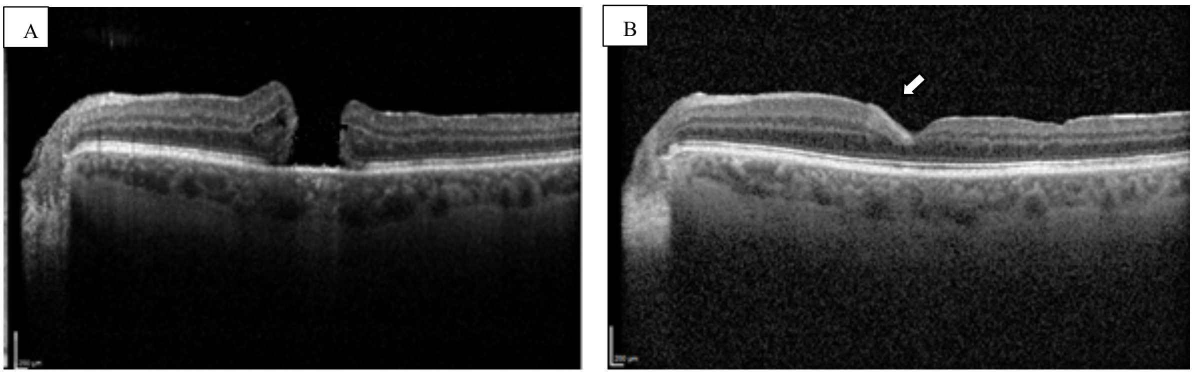


Case 18. (A) Full thickness macular hole successfully treated with LAM graft (B). Note the LAM in B (white arrow in B).

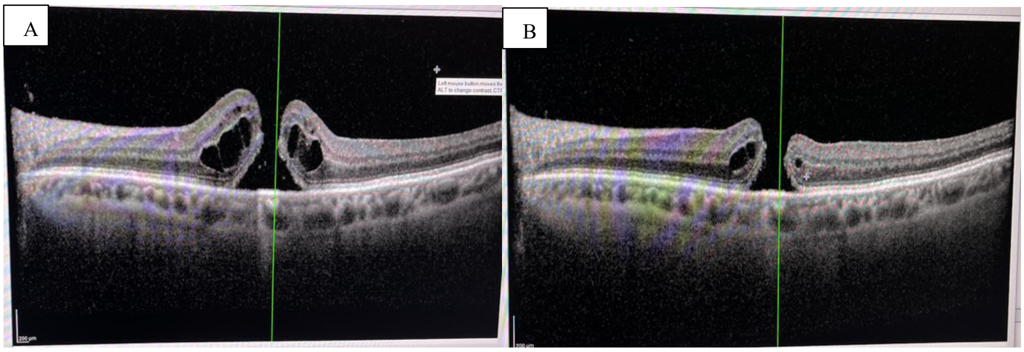


Case 19. Full thickness macular hole unsuccessfully treated with LAM graft (B) due to dislocation of the LAM, into the peripheral retina.
